# Development of protective immunity against African swine fever depends on host-environment interactions

**DOI:** 10.3389/fvets.2025.1553310

**Published:** 2025-06-10

**Authors:** Emilia Radulovic, Kemal Mehinagic, Tsering Monika Wüthrich, Markus Hilty, Artur Summerfield, Nicolas Ruggli, Charaf Benarafa

**Affiliations:** ^1^Institute of Virology and Immunology IVI, Mittelhäusern, Switzerland; ^2^Department of Infectious Diseases and Pathobiology, Vetsuisse Faculty, University of Bern, Bern, Switzerland; ^3^Graduate School for Cellular and Biomedical Sciences, University of Bern, Bern, Switzerland; ^4^Institute for Infectious Diseases, University of Bern, Bern, Switzerland; ^5^Multidisciplinary Center for Infectious Diseases (MCID), University of Bern, Bern, Switzerland

**Keywords:** African swine fever, microbiota, specific pathogen-free (SPF) pigs, *in vivo*, immune response, resilience

## Abstract

African swine fever virus (ASFV) is a major threat for pig health and meat production in many countries. The development and commercialization of vaccine candidates are complicated by efficacy and safety concerns. Improved vaccine design requires further studies to identify factors that regulate immune responses to vaccines leading to protective immunity against a virulent challenge. In a previous study, we reported that infection with the moderately virulent ASFV field strain Estonia 2014 was less severe in specific pathogen-free (SPF) pigs than in conventional farm pigs, which differ in their gut microbiome and their basal immune activation status. As shown previously using intramuscular infection, SPF pigs were more resilient to oronasal infection with the ASFV Estonia 2014 strain compared to farm pigs, which showed increased fever and clinical signs. All SPF and farm pigs nevertheless survived the infection and remained viremic for approximately 4 months. When all animals had no detectable viremia, both groups were rechallenged with the virulent ASFV Armenia 2008 strain. SPF pigs were fully protected against disease and showed little or no viremia upon re-challenge. In contrast, farm pigs developed high viremia, high proinflammatory cytokine responses, severe clinical signs, and 40% (2 of 5 pigs) reached humane endpoints. Our findings suggest that limited prior immune exposure to other pathogens and/or the microbiome composition of SPF pigs promotes resilience to infection with a moderately virulent strain such as Estonia 2014, and importantly promotes the development of a strong protective immune response against a second challenge with a virulent ASFV strain. In conclusion, testing safety and efficacy of live attenuated vaccine candidates should take into account the specific hygiene conditions and the associated changes of general immune status of pigs in clinical trials.

## Introduction

African swine fever virus (ASFV) causes a severe hemorrhagic fever in domestic pigs and wild boars. ASFV was first described in Africa (1), where it continues to cause sporadic outbreaks in domestic pigs (2). Since 2007, a highly virulent genotype II strain is the cause of the largest recorded outbreak of African swine fever (ASF) that is now a global challenge (3–5). Depending on the strain virulence, the age of the animals, and the infectious dose received, infected pigs can die suddenly without showing any prior symptoms due to severe internal bleeding, organ failure, or other complications. Frequently, ASF presents as an acute disease with skin hyperemia, petechiae, and hemorrhagic diarrhea accompanying the febrile symptoms, with death occurring within 7–10 days (6).

To effectively combat ASFV and mitigate its impact, region-specific and global control strategies must be implement including robust disease surveillance, biosecurity measures in farms, implementation of control zones, movement and trade restrictions of animals and meat products, and global cooperation addressing both domestic and wild swine populations (2, 7). An effective and safe vaccine against ASFV would be an integral part of the global strategy against this disease if it were to become available. However, few experimental vaccines induce protection against an experimental challenge and the mechanisms associated with protection are not well defined. Live attenuated vaccine (LAV) candidates that are based on deletion of putative virulence factors have shown efficacy against homologous challenge, but concerns about residual pathogenicity, virulence reversion, recombination with circulating strains, and lack of a DIVA marker are major hurdles for the licensing of LAV candidates (8–10). In addition, at higher doses LAVs have been reported to induce clinical disease and, paradoxically can also be associated with a reduced rate of protection (11).

We have previously shown that pigs raised under specific pathogen-free (SPF) conditions at the Institute of Virology and Immunology (IVI) or raised in a conventional pig farm are equally highly susceptible to an intramuscular challenge with the virulent ASFV Armenia 2008 strain and succumb to the disease within 6 days. In contrast, the SPF pigs developed a markedly milder and shorter disease form of ASF than the farm pigs following intramuscular infection with the moderately virulent ASFV Estonia 2014 strain (12). Profound differences in gut microbiota composition were observed between the two groups, and the SPF pigs had a lower steady state body temperature than the farm pigs. Importantly, the baseline immune status of the SPF pigs was characterized by a lower level of inflammatory and immune activation and the SPF pigs had lower numbers of circulating immune cells, particularly neutrophils and lymphocytes. Blood cell transcriptomic profiles prior to infection revealed that the farm pigs had upregulated transcriptional modules associated with immune cell proliferation, pro-inflammatory pathways, dendritic cell activation, type I interferon signaling, and lymphocyte proliferation. These findings indicate, not surprisingly, that farm pigs are exposed to a more immune-activating environment including commensals, pathogens, and their metabolites compared to our SPF pigs. Here, we set out to investigate whether prior environmental exposure and the associated changes in the immune system in SPF and farm pigs had an impact on the development of a protective immunity induced by an oronasal infection with ASFV Estonia 2014 that was tested with a subsequent lethal oronasal challenge with ASFV Armenia 2008.

## Materials and methods

### Animals

Thirteen male (castrated) and female Large White domestic pigs, 10–11 week-old with a body weight of 20–25 kg were obtained from the IVI SPF breeding facility (*n* = 5) or a local farm (*n* = 8). The pigs from the IVI SPF facility are not vaccinated and are negative for the porcine viral and bacterial pathogens listed in Table 1. Access to the SPF facility is restricted to animal caretakers and includes a showering airlock at the entrance. The SPF facility is supplied with filtered air at positive pressure and pigs are fed X-Ray-irradiated (>10 kGy) pig pellet diet (Granovit AG, KLIBA NAFAG, Kaiseraugst, Switzerland) and autoclaved hay and straw. No animal has been introduced in the facility since the initial colonization in 1993 and no clinical signs of infectious disease (diarrhea, abortion, respiratory symptoms) were observed during this period. The genetic pool is maintained at a similar status as current Swiss/European Large White pig farms *via* artificial insemination using semen from SUISAG (Sempach, Switzerland). The commercial farm pigs were from a high standard breeding and fattening pig farm and were vaccinated against porcine circovirus 2, *Escherichia coli*, and *Lawsonia intracellularis*. The SPF and farm pigs were housed in the BSL-3Ag containment facilities for the whole duration of the study. During the trials, all animals were fed a commercial pig pellet diet with hay supplementation and water *ad libitum*. The animals were maintained in a 13:11 h light cycle. Infections, body temperature measurements, clinical scores, and sampling were performed at Zeitgeber time ZT 2-4. The animals were euthanized by electrical stunning followed by exsanguination.

**Table 1:** List of pathogens excluded from the IVI SPF pigs.

**Viruses**
Transmissible gastroenteritis virus (TGEV)
Porcine epidemic diarrhea virus (PEDV)
Porcine respiratory coronavirus (PRCV)
Classical swine fever virus (CSFV)
African swine fever virus (ASFV)
Porcine reproductive and respiratory syndrome virus (PRRSV)
Foot-and-mouth disease virus (FMDV)
Porcine reproductive and respiratory syndrome virus (SVD)
Suid herpesvirus (SuHV1)
Porcine parvovirus (PPV)
Swine influenza A virus (SIV A)
Porcine circovirus type 2 (PCV-2)
**Bacteria**
*Actinobacillus pleuropneumoniae*
*Mycoplasma hyopneumoniae*
*Streptococcus suis*
*Haemophilus parasuis*
*Erysipelothrix rhusiopathiae*
*Bordetella bronchiseptica*
*Pasteurella multocida*
*Lawsonia intracellularis*
*Chlamydia* sp.
*Brucella* sp.
*Leptospira* sp.

### ASFV infections *in vivo*

**Figure 1:**
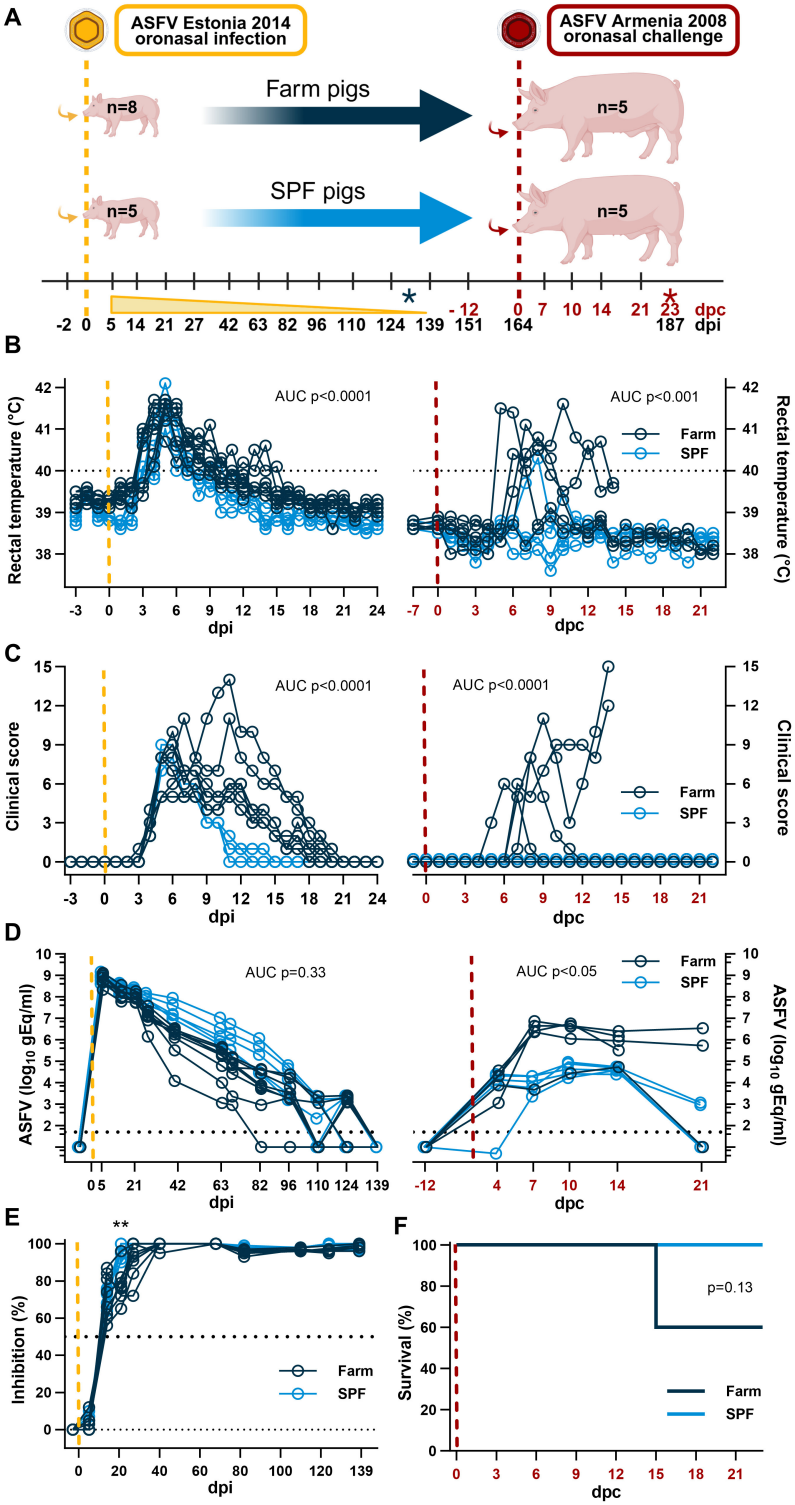
Experimental design and clinical parameters. **(A)** Diagram of experimental setup. SPF and farm pigs were inoculated oronasally with the ASFV Estonia 2014 (yellow) and re-challenged oronasally 164 days later with the Armenia 2008 strain (red). Blood samples were taken at the indicated time points and clinical parameters were measured daily in the first 3 weeks after each infection and once a week thereafter. The time points indicated in black refer to days post infection (dpi) with the Estonia 2014 strain infection; the time points in red refer to days post challenge (dpc) with the Armenia 2008 strain. The black asterisk indicates euthanasia of 3 farm pigs in excess before the second challenge. The red asterisk indicated the end of the experimentation after the second challenge. **(B)** Rectal temperature. **(C)** Clinical score. **(D)** Virus copy numbers determined in EDTA blood by qPCR. **(E)** Seroconversion determined using a competitive anti-p72 ELISA after the ASFV Estonia 2014 strain infection. **(F)** Survival curve after the Armenia 2008 strain challenge. **(A–F)** Yellow dashed lines represent the inoculation with the ASFV Estonia 2014 strain; the red dashed lines represent the inoculation with the ASFV Armenia 2008 strain. **(B–D)** Differences between groups were analyzed by unpaired *t*-test comparing the area under the curve (AUC) between the two groups after each infection. **(E)** Differences in antibody levels were analyzed by two-way ANOVA (mixed model) with Sidak's multiple comparisons test; **p < 0.01. **(F)** Differences in survival were analyzed by Log-rank (Mantel-Cox) analysis. **(A)** Created in BioRender. https://BioRender.com/5wxbb8e.

The SPF and farm pigs were acclimatized for 5 days in two separate BSL-3Ag stables. The pigs were infected oronasally with ~5 × 10^9^ genome equivalents (gEq) using a syringe with a 5 cm rubber tubing. The inoculum was sprayed equally in one nostril (2.5 ml) and in the back of the mouth (2.5 ml) while the head of the animal was held up. The ASFV Estonia 2014 strain inoculum was a pig blood sample from a previous experiment (12) containing ~1 × 10^9^ gEq/ml determined by qPCR as described below. When viremia was no longer detectable by qPCR, five surviving pigs of each group were re-challenged oronasally with ~10^6^ TCID_50_ of the highly virulent ASFV Armenia 2008 at 164 days post immunization (dpi), which corresponds to 0 day post-challenge (dpc) (Figure 1A). A larger group of farm pigs (*n* = 8) than SPF pigs (*n* = 5) was used because we anticipated some lethality in this group after the first infection. However, all animals from both groups survived and three farm pigs were euthanized prior to the second infection to match the number of SPF pigs. Due to the low number of animals, we euthanized three farm pigs (#3, #6, #8) which were representative of the group including both moderate and high clinical scores after the first infection.

Body temperature and clinical parameters were assessed daily by veterinarians (KM, NR, CB) based on an adapted clinical score checklist described previously for experimental classical swine fever virus infections (13). The re-challenged pigs were euthanized at the latest on 187 dpi (corresponding to 23 dpc) unless discontinuation criteria were reached before. A cumulative score of 18 and/or a single score of 3 in any of the following parameters: liveliness, body tension, breathing, walking, or skin, were defined as the humane endpoints for discontinuation of the experiment for each pig. Blood samples were collected 1–3 days prior to infection and on 5, 14, 21, 27, 40, 63, 68, 82, 96, 110, 124, 139, 151, 168 (4 dpc), 171 (7 dpc), 174 (10 dpc), 178 (14 dpc), and 185 dpi (21 dpc), or until discontinuation criteria were reached. A full necropsy was performed on all animals; whole blood (EDTA), serum, and organs were collected for virus quantification and titration.

### Virus stocks and quantification

The original stocks of genotype II ASFV strains Estonia 2014 (Genbank accession number LS478113.1) and Armenia 2008 were generously provided by Sandra Blome and Martin Beer, Friedrich-Loeffler-Institut, Greifswald-Insel Riems, Germany. Virus titration was determined by indirect immunofluorescence assay in immortalized porcine kidney macrophage (IPKM) cells (14), a generous gift of Takehiro Kokuho, National Agriculture and Food Research Organization (NARO), Tokyo, Japan. Positive cells were counted, and the titer was calculated using the Reed-Muench method.

For qPCR, DNA was extracted using the NucleoMag VET kit (Macherey-Nagel) and the KingFisher extraction platform following manufacturers' instructions. All nucleic acid extractions were performed with 200 μl of either serum, whole blood, or organ homogenates in RA1 lysis buffer adjusted to contain 5 mg of tissue. Subsequently, qPCR for the B646L gene (p72) was performed in triplicates for samples and standards to determine genome equivalents (gEq) as described previously (12).

### Hematology and flow cytometry

Differential blood cell counts were determined from EDTA blood samples using an automated hematology analyzer (VetScan HM5, Abaxis). The percentage of leukocyte subsets were determined by flow cytometry and absolute subset counts were calculated using white blood cell (WBC) values from the hematology analyzer. Whole blood (100 μl) was incubated with two sets of antibodies for myeloid and lymphocyte subsets. Data acquisition (100,000 single-cell events) was done on a BD FACS Canto II (BD Bioscience), and data analyzed with FlowJo v10. The gating strategy is described in Supplementary Figure 1. In brief, single cells were determined by forward scatter (FSC) and side scatter (SSC) parameters. Neutrophils were identified as CD172+SSC^hi^ and monocytes as CD14^+^CD172a^+^. In the lymphocyte panel SCC^low^ cells were gated as NK cells (CD16^+^) and T cells (CD3^+^). T cell subsets were first gated as cytotoxic T cells (CD4^−^CD8β^+^), then CD8β^−^ T cells gated in four subsets based on CD4 and CD8α. CD4^+^CD8α^−^ are naïve helper αβ T cells; CD4^+^CD8α^+^ are principally effector/memory helper T cells; CD4^−^CD8α^−^ and CD4^−^CD8α^+^ include γδ T cells and αβ T cells with less well defined functional phenotypes. The antibodies used are listed in Table 2.

**Table 2:** List of antibodies.

**Target**	**Clone**	**Isotype**	**Supplier**	**Cat#**	**Labels**
CD3	BB23-8E6-8C8	IgG2a	BD	561478	PerCP-Cy5.5
CD4	74-12-4	IgG2b	BD	559586	PE
CD8α	76-2-11	IgG2a	IVI	Hybridoma	-
CD8β	PPT23	IgG1	Bio-Rad	MCA5954GA	-
CD14	322A-1 My4	IgG2b	Coulter	6603511	FITC
CD16	G7	IgG1	LSBio	LS-C21674	FITC
CD172a	74-22-15A	IgG2b	IVI	Hybridoma	-
Mouse IgG1			BioLegend	406613	PE-Cy7
Mouse IgG2a			BioConcept	1082-08	Biotin
Mouse IgG2b			ThermoFisher	A-21242	AF647
Biotin		SA	BD	561419	V500

### Cytokine and antibody measurements

Serum cytokines were determined using a custom premixed Milliplex Map porcine cytokine/chemokine magnetic beads kit (Millipore, USA) for the chemokine IL-8 and nine cytokines (IL-1α, IL-1β, IL-1ra, IL-2, IL-4, IL-6, IL-10, IL-12, IL-18). Antibodies against ASFV protein p72 were detected using INgezim PPA COMPAC blocking ELISA Kit (R.11.PPA.K.3, Ingenasa, Madrid, Spain) according to manufacturer's instruction. The results are expressed in % of inhibition, with following cut-off values: < 40%, negative; 40–50%, doubtful; ≥50%, positive. Serum samples were tested in duplicate.

### Fecal microbiota analysis

Fecal samples were collected from SPF and farm pigs prior to infection, 1 week after acclimatization into the biocontainment stables of the IVI. Stool collection was performed using sterile swabs and containers and stored at −80°C. DNA was extracted using the QIAamp Fast DNA Stool Mini Kit following the manufacturer's guidelines. For an optimal lysis of and separation of impurities from stool samples, the stools were first suspended and vortexed in 1 ml of InhibitEX Buffer. The V4 region of the 16S rRNA gene was amplified using forward (5'-GTGCCAGCMGCCGCGGTAA-3') and reverse (5'-GGACTACHVGGGTWTCTAAT-3') primers modified with an Illumina adaptor sequence at the 5′ end. PCR products were purified using the QIAquick PCR Purification Kit (Qiagen, Hilden, Germany). Samples were passed through to a MiSeq Illumina sequencing platform for indexing and paired-end sequencing (2 × 250 bp; reagent kit, v2). Sequencing data were analyzed using the DADA2 package (version 1.16.0) in R software (version 4.0.2) for the identification of amplicon sequence variants (ASV). The taxonomy assignment of the ASVs was done using the SILVA (version 132) database. Contaminating sequences were identified using the decontam package (version 1.8.0) in R. Contaminants were identified by their frequency of occurrence and independently within each batch. Distance matrices were calculated for the beta-diversity analyses and used as input files for the non-metric multidimensional scaling (NMDS) plots. Statistical analysis was performed by permutation test (PERMANOVA; Adonis function).

### Statistical analysis

Statistical analyses were performed using GraphPad Prism version 8.0.0 for Windows unless otherwise indicated. The number of samples and statistical tests used are indicated in figure legends.

## Results

We showed previously that SPF pigs develop a milder disease and have a better survival rate than farm pigs following an intramuscular inoculation of the attenuated Estonia 2014 ASFV strain (12). The current study was designed with two phases. In the first phase, 5 SPF and 8 farm pigs were inoculated oronasally with the Estonia 2014 strain to validate our previous findings where the intramuscular route was used for infection. Blood samples were taken at regular intervals to determine the duration of viremia between pigs of the two groups. In the second phase of the study, 5 pigs from each group were challenged with the highly virulent Armenia 2008 ASFV strain to determine (1) whether prior exposure to Estonia 2014 provides immunity against a challenge with Armenia 2008; and (2) whether SPF and farm pigs have a similar response to this virulent challenge (Figure 1A).

Oronasal inoculation 5 × 10^9^ gEq of ASFV Estonia 2014 induced a first wave of clinical disease with a peak on 5–7 dpi characterized by high fever, loss of appetite, and reduced liveliness. SPF pigs presented significantly milder disease in intensity and duration than farm pigs. All SPF pigs fully recovered by 15 dpi, whereas farm pigs experienced a second peak of clinical signs and a delayed recovery until 21 dpi (Figures 1B, C, left panels). Thus, farm pigs showed a significantly extended duration of clinical signs with fever, weakness, apathy, and reduced appetite. These data concord with our previous study where the animals were inoculated intramuscularly (12). A notable difference was the absence of lethality in the farm pig group, while in the previous study we had observed 50% lethality in that group after intramuscular inoculation of the Estonia 2014 strain. The highest virus loads at ~10^9^ gEq/ml of whole blood were measured in the first sample taken on 5 dpi and levels gradually diminished over time at a similar rate for the two groups until no virus was detectable by PCR in the blood of all the animals on 139 dpi (Figure 1D, left panel). Using a competitive anti-p72 ELISA, seroconversion (>50% inhibition) was observed in all animals at 14 dpi, and maximal antibody responses (100% inhibition) were reached sooner in SPF pigs (27–40 dpi) than in farm pigs (63 dpi) (Figure 1E).

Prior to the second phase of the study, 3 pigs from the farm group were euthanized on 131 dpi to have the same number of pigs in each group. The remaining animals (*n* = 5/group) were challenged oronasally with 10^6^ TCID_50_ of the highly virulent Armenia 2008 ASFV strain on 164 dpi that is 0 day post challenge (dpc) (Figure 1A). Strikingly, SPF pigs showed no apparent clinical signs and maintained normal liveliness and appetite after challenge and only one SPF pig had fever for two consecutive days (39.7 and 40.3 on 7 and 8 dpc, respectively). In contrast, all farm pigs displayed severe clinical signs with high fever (>40°C), apathy, weakness, inability to stand, loss of appetite, emaciation, and skin petechiae (Figures 1B, C, right panels). Two farm pigs were euthanized on 14 dpc after reaching the predetermined maximal clinical scores (Figure 1F). Viremia was significantly higher in the EDTA blood of farm pigs compared to SPF pigs (Figure 1D, right panel). The viremia reached a significantly lower plateau at 4 dpc in SPF pigs and at 7 dpc in farm pigs. At the end of the study on 21 dpc, all five SPF pigs and one farm pig had low or no detectable virus in blood, whereas the two remaining farm pigs still had a relatively high viremia (Figure 1D). These results indicate that pigs surviving an infection with the attenuated Estonia 2014 ASFV strain can develop a protective response against a lethal challenge with the highly pathogenic ASFV Armenia 2008 strain. Furthermore, the SPF status provides stronger protection against clinical disease and better control of the viremia compared to conventional farm pigs.

The fecal microbiome of each animal was analyzed prior to the first infection with the Estonia 2014 ASFV strain. While the diversity was similar, the microbiome composition was significantly different between SPF and farm pigs: *Prevotellaceae* were the dominant family in farm pigs, whereas *Bacteroidaceae* and *Muribaculaceae* were the most abundant in SPF pigs (Supplementary Figure 2A). We found that the bacterial family composition remained highly consistent for each group of this study compared to our two previous studies (12) that were collected over a year before. These consistent findings were largely expected for the pigs from the IVI SPF facility where hygiene control is strict, but finding a stable microbiome was less expected for animals from a commercial pig farm (Supplementary Figure 2A).

**Figure 2:**
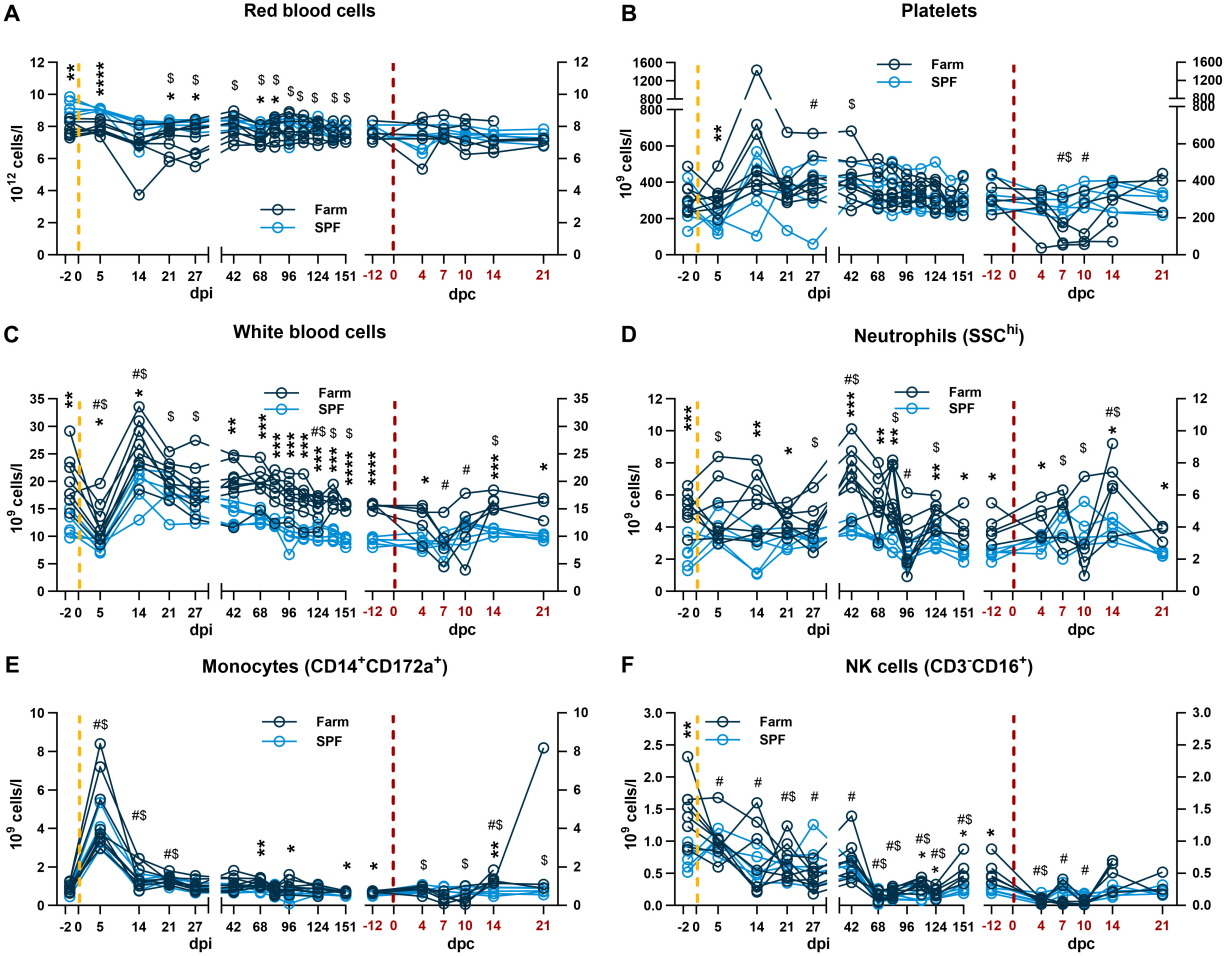
Blood and innate immune cell count profiles. Each panel is shown in two parts that were analyzed independently. On the left, the yellow dashed line represents the inoculation with the ASFV Estonia 2014 strain and time points are referred to as days post infection (dpi); on the right, the red dashed line represents the challenge with the ASFV Armenia 2008 strain and time points are referred to as days post challenge (dpc, in red). **(A–C)** Red blood cell, platelet, and white blood cell (WBC) counts were determined in whole blood. **(D–F)** Absolute counts of **(D)** neutrophils, **(E)** monocytes, **(F)** and NK cells. The percentage of each subset was determined by flow cytometry and absolute numbers were calculated using WBC counts. **(A–F)** Data points represent values for individual pigs. Differences between SPF and farm groups were analyzed by two-way ANOVA (mixed model) with Sidak's multiple comparisons test. Statistical differences between groups are shown as *p < 0.05; **p < 0.01; ***p < 0.001; ****p < 0.0001. Changes in cell counts within each group were also compared to their respective baseline values taken before each infection at −2 dpi or at −12 dpc (equivalent to 151 dpi). Significant differences within groups compared to their respective pre-challenge status are indicated for SPF ($, *p* < 0.05) and farm pigs (#, *p* < 0.05); data were analyzed using mixed-effects analysis.

**Figure 3:**
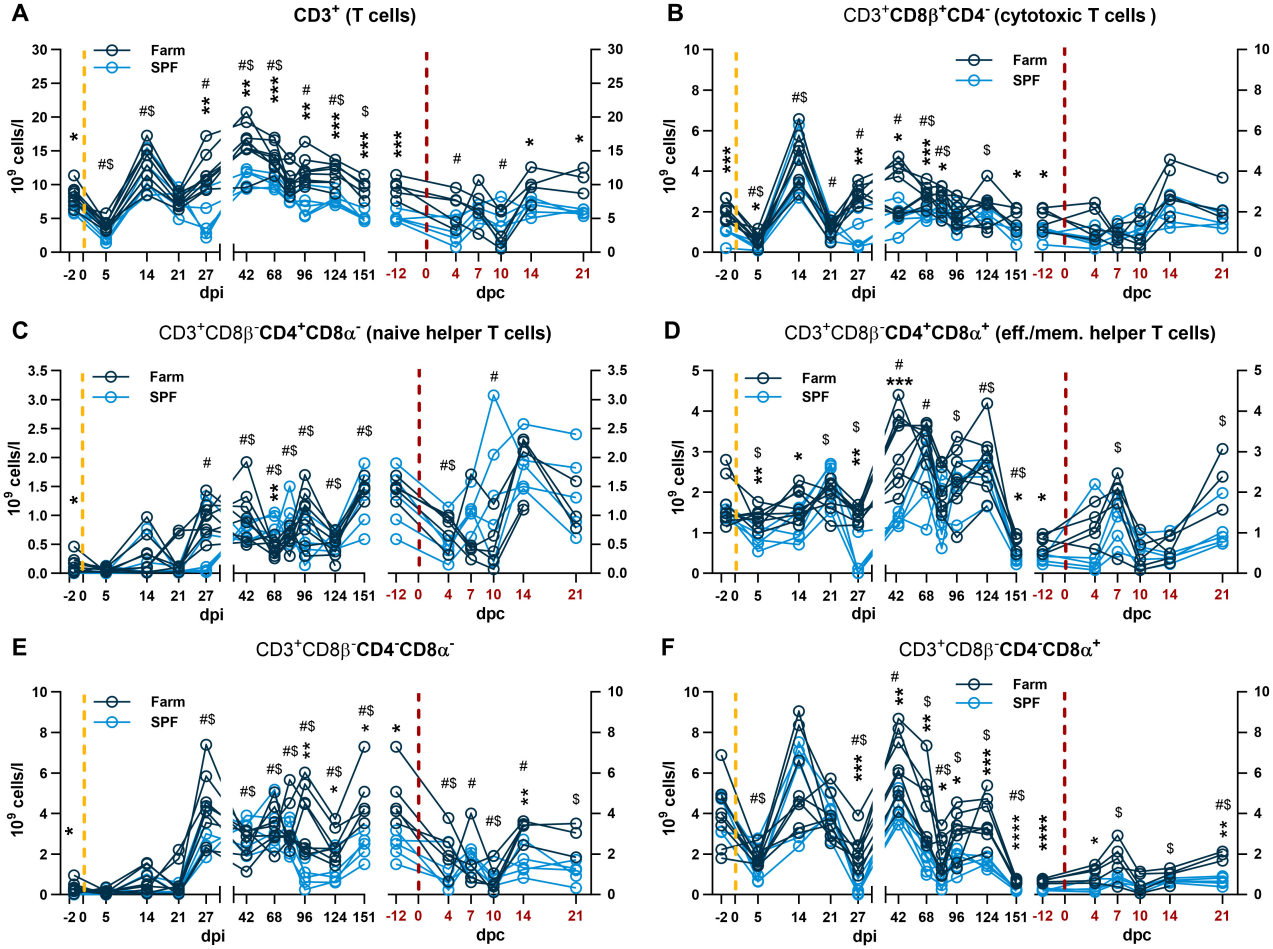
Blood T cell subset analysis. Each panel is shown in two parts that were analyzed independently. On the left, the yellow dashed line represents the inoculation with the ASFV Estonia 2014 strain and time points are referred to as days post infection (dpi); on the right, the red dashed line represents the challenge with the ASFV Armenia 2008 strain and time points are referred to as days post challenge (dpc, in red). **(A)** Total CD3^+^ T cell counts. **(B)** Cytotoxic T cells defined by CD3^+^CD4^−^CD8β^+^. **(C–F)** After gating cytotoxic T cells, the remaining T cells (CD3^+^ CD8β^−^) were identified by relative expression of CD4 and CD8α as **(C)** naïve helper T cells CD8β^−^CD4^+^CD8α^−^, **(D)** effector and memory (eff./mem.) T cells CD8β^−^CD4^+^CD8α^−^; **(E)** CD8β^−^CD4^−^CD8α^−^, and **(F)** CD8β^−^CD4^−^CD8α^+^. **(A–F)** The percentage of each subset was determined by flow cytometry and absolute numbers were calculated using WBC counts. **(A–F)** Data points represent values for individual pigs. Differences between SPF and farm groups were analyzed by two-way ANOVA (mixed model) with Sidak's multiple comparisons test. Statistical differences between groups are shown as *p < 0.05; **p < 0.01; ***p < 0.001; ****p < 0.0001. Changes in cell counts within each group were also compared to their respective baseline values taken before each infection at −2 dpi or at −12 dpc (equivalent to 151 dpi). Significant differences within groups compared to their respective pre-challenge status are indicated for SPF pigs ($, *p* < 0.05) and farm pigs (#, *p* < 0.05); data were analyzed using mixed-effects analysis.

Analysis of circulating blood cells at baseline prior to the first infection with the Estonia 2014 ASFV strain showed significantly higher red blood cell (RBC) and lower white blood cell (WBC) counts in SPF compared to farm pigs at baseline (Figures 2A, C). No significant difference in platelet (PLT) counts was noted at baseline (Figure 2B). Flow cytometry analysis at baseline revealed that the low WBC counts in SPF pigs were caused by reduced neutrophils, NK cells, and subsets of T cells such as cytotoxic CD8β+ T cells (Figures 2D, F, 3). These data are consistent with our previous study that revealed a more naïve immune system in SPF pigs with particularly low neutrophil and other cell subset counts in blood and are consistent with the different hygienic status and the stable microbiome data over time in both groups (12).

After infection with the Estonia 2014 strain, a sharp drop in WBC counts at 5 dpi followed by a rebound at 14 dpi were observed in both groups (Figure 2C). The effect at 5 dpi was driven by lymphopenia with a reduction of total T cells and particularly CD8β^+^ and CD8β^−^CD4^−^CD8α^+^ in both groups (Figures 3A, B, F), and NK cells in farm pigs (Figure 2F). This lymphopenia was followed by a sharp increase of T cells, in particular cytotoxic CD8β^+^ T cells at 14 dpi (Figures 3A, B). Absolute counts of monocytes sharply peaked in both groups at 5 dpi and remained elevated on 14 and 21 dpi before returning to baseline from 27 dpi (Figure 2E).

During the recovery phase, where both groups remained viremic but asymptomatic, differences in total WBC remained comparable to the baseline prior to infection with significantly higher leukocyte counts in farm pigs compared to SPF pigs (Figure 2C). As for the baseline, neutrophil, NK cell and T cell counts significantly contributed to the reduced WBC in SPF pigs compared to farm pigs (Figures 2, 3). Prior to the challenge with the Armenia 2008 strain (-12 dpc, equivalent to 151 dpi), a second baseline was established for RBC, PLT and leukocyte subset counts (Figures 2, 3, right panels). RBC and PLT counts were similar in both groups, while WBC counts were lower in the SPF group. Neutrophil, monocyte, NK cell, and T cell subset counts were all significantly lower in the SPF pigs compared to farm pigs at −12 dpc.

After the challenge with the ASFV Armenia 2008 strain, we observed significant reduction of platelets and white blood cell counts in farm pigs, consistent with acute ASF disease (Figures 2B, C, right panels). The loss of total T cells was also prominent on 4–10 dpc in farm pigs, which was observed in several T cell subsets, but no clear pattern could be identified in relation clinical severity (Figure 3). In contrast, the challenge did not induce leukopenia in the SPF group. On the contrary, we observed increased neutrophils on 7–14 dpc, monocytes on 4–21 dpc (Figures 2D, E, right panels), and CD4^+^CD8α^+^ effector/memory helper T cells on 7 dpc (Figure 3D, right panel).

**Figure 4:**
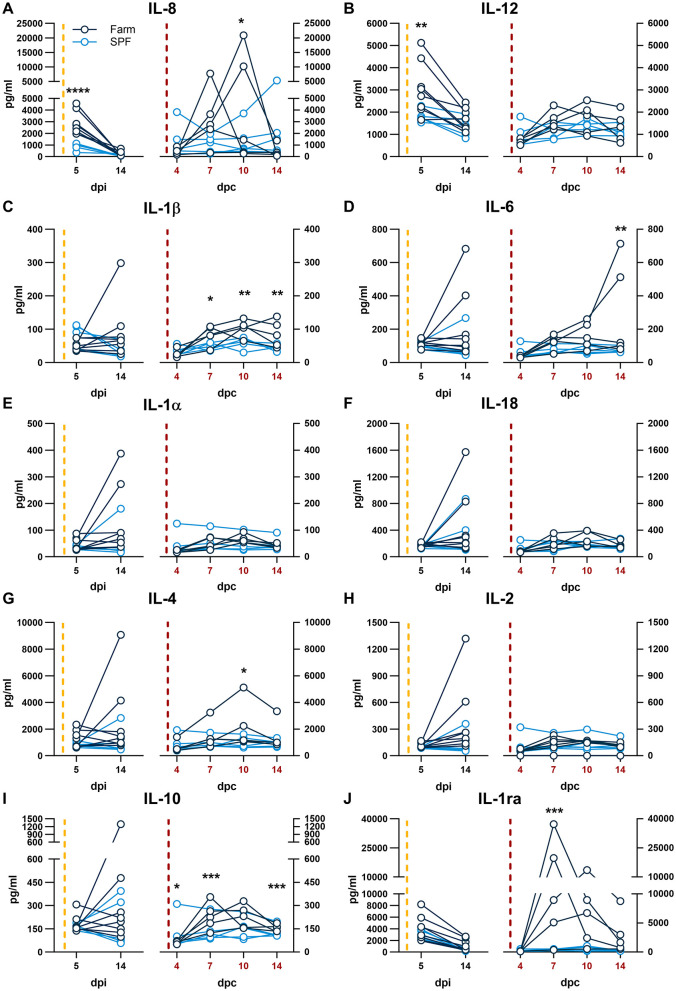
**(A–J)** Serum cytokines. Each panel is shown in two parts that were analyzed independently. On the left, the yellow dashed line represents the inoculation with the ASFV Estonia 2014 strain and time points are referred to as days post infection (dpi); on the right, the red dashed line represents the challenge with the ASFV Armenia 2008 strain and time points are referred to as days post challenge (dpc, in red). Cytokine levels were determined by multiplex ELISA. Differences between SPF and farm groups were analyzed by two-way ANOVA (mixed model); *p < 0.05, **p < 0.01, ***p < 0.001, ****p < 0.0001.

The cytokine response in serum was evaluated 5 and 14 dpi with the Estonia 2014 strain and 4, 7, 10, 14 dpc with the Armenia 2008 strain. At 5 dpi of the first infection, only the neutrophil chemokine IL-8 and the pro-inflammatory cytokine IL-12 were significantly higher in farm compared to SPF pigs (Figures 4A, B, left panels). Upon challenge with the Armenia 2008 strain, significantly higher levels of IL-8, pro-inflammatory cytokines IL-1β and IL-6, and the Th2 cytokine IL-4 were significantly increased in the farm pigs compared to SPF pigs at one or several time points after the Armenia 2008 strain challenge (Figure 4). At 7 dpc, farm pigs had significantly elevated levels of the anti-inflammatory cytokine IL-10 and the IL-1 receptor antagonist (IL-1ra) (Figures 4I, J), and this increase was particularly high in the farm pigs with the most severe clinical scores, suggesting regulatory feedback. In the SPF pig group, the cytokine levels were not altered in response to the challenge with Armenia 2008 strain. We also examined the changes in fecal microbiota of the two pig groups on 10 dpc. NMDS analysis showed that SPF and farm pigs clustered differently from each other at baseline (−2dpi) and at 10 dpc (Supplementary Figure 2B).

Overall, the lack of a strong leukocyte depletion and cytokine response in SPF pigs associated with a very low-grade viremia and clinical disease following the ASFV Armenia 2008 strain challenge demonstrates that SPF pigs have acquired a strong and almost sterilizing immunity against ASFV.

## Discussion

In this new study comparing piglets born and raised in our SPF facility with those from a conventional commercial farm, we found that the gut microbiomes were strikingly different in composition as reported previously (12). The main bacterial families of the gut microbiome of pigs of the respective groups were also identical to the samples evaluated from the same facilities more than a year before. This constancy indicates that the microbiome is a stable component of the animals from a specific facility whether it is an experimental SPF facility, or a commercial pig farm, where environmental conditions may not be as strictly controlled. In addition, the farm pigs also had higher counts of circulating leukocytes and higher body temperature at baseline compared to SPF pigs as shown previously.

Furthermore, we showed a higher resilience of SPF pigs compared to farm pigs following oronasal infection with the moderately virulent ASFV Estonia 2014 strain, thus confirming our previous findings following intramuscular infection with this strain (12). In both studies, SPF pigs demonstrated significantly less severe clinical signs and a faster recovery than farm pigs. A notable difference was that we had observed 50% lethality for the farm pigs when the pigs were infected intramuscularly with ASFV Estonia 2014 strain in the previous study, whereas here, all farm pigs ultimately recovered from the infection after oronasal infection with the same inoculum dose. The improved outcome in farm pigs may be explained by the different route of infection, with more severe outcome after intramuscular infection. In addition, only two blood samples were taken in the acute phase of the disease on 5 and 14 dpi in this study, whereas seven blood samples were taken in the previous study on 1, 2, 4, 5, 7, 11, and 14 dpi, which may have compounded the effects of the virus-induced thrombocytopenia and contributed in part to the lethality.

The viremia after oronasal instillation of the ASFV Estonia 2014 strain was followed until it became undetectable by PCR in serum and whole blood. SPF and farm groups had comparable virus load at all time points. The highest viremia was measured at the first sampling at 5 dpi in both groups and the virus titers gradually reduced over several months until no virus was detectable by PCR on 139 dpi in all animals. This prolonged viremia is consistent with previous publications (15), notably the kinetics of the viremia reported for this strain (16). These findings indicate that the more severe disease observed in farm pigs is not associated with uncontrolled virus replication compared to SPF pigs and that other mechanisms linked to the host response are driving the pathology.

Upon subsequent challenge with the ASFV Armenia 2008 strain, the almost complete absence of clinical signs and the controlled viremia in SPF pigs contrasted with the more severe disease in farm pigs including the euthanasia of 2 out of 5 farm pigs. Upon challenge, farm pigs showed significantly higher viremia, increased inflammatory cytokine release, and a characteristic transient drop in NK cell and T cells counts in blood. These findings indicate that SPF pigs developed a stronger protective adaptive immune response and that host immune factors influenced by the housing conditions and potentially the microbiome are also crucially important for the development of a strong protective adaptive immunity. The timing of the seroconversion against the major capsid antigen p72 indicated a slight delay in farm pigs. Nevertheless, this parameter is unlikely an indicator of protection against a re-challenge as all animals raised a strong antibody response against this antigen.

The pattern of cytokine expression analysis indicates an association between high levels of the neutrophil chemokine IL-8 with higher clinical scores after infection with either strains. Furthermore, higher IL-8 responses at 5 dpi during the first infection with the ASFV Estonia 2014 strain also correlated with a worse outcome in farm pigs after the rechallenge months later with the Armenia 2008 strain. This suggests that the lower basal immune activation status of SPF pigs leads to lower secretion of IL-8 and milder clinical symptoms after the first infection but enhanced adaptive responses after re-challenge. The fatal outcome in the 2 farm pigs was further associated with very high levels of the inflammatory cytokine IL-6 at 14 dpc, which is consistent with previous studies (12, 17). These data are consistent with evidence indicating that high levels of serum IL-6 are associated with a bad prognosis in severe infections (18, 19).

We had previously shown that after infection with the ASFV Estonia 2014 strain, SPF pigs expressed significantly higher levels of IL-1ra at 4 dpi. IL-1ra blocks signaling of the receptor for the inflammatory cytokines IL-1α and IL-1β. We speculated that an early and high peak of IL-1ra may be a protective factor associated with a higher resilience to a moderately virulent ASFV infection. Here, the first sample taken after infection with the Estonia 2014 strain was performed only at 5 dpi, which prevented us to make a direct comparison. Like in the previous study, we found no significant difference in IL-1ra levels between the two groups at 5 dpi. The interpretation of the results of the current study is limited by the relatively low number of animals per group and the lack of cytokine data prior to each infection. Future work should include the validation of early innate markers associated with resilience.

The analysis of the blood cell subset dynamics in this longitudinal study did not provide a strong correlation between responses after the infection with Estonia 2014 that could provide a predictive outcome following the challenge with the virulent ASFV strain. However, we observed a significant increase in CD4^+^CD8α^+^ T cells in SPF pigs, suggesting the expansion of effector and/or memory T cells associated with protection. Further studies are needed to explore antigen-specific T cell responses and functional antibody assays correlating with protection.

This study highlights again the importance of environmental factors and hygiene status such as prior infections and microbiota composition on innate and adaptive immune responses in pigs. It remains unclear if the differences in resilience and adaptive responses between SPF and farm pigs are directly linked with beneficial or opportunistic bacteria and their metabolites, or if this is only a bystander correlation. Fecal microbiota transfers studies may help understand the function of the microbiota in the mechanisms of resilience and protective immune responses against ASFV.

In conclusion, controlled studies of pigs with different hygienic backgrounds provide a solid platform to explore the mechanism and correlates of protections against ASF, and how they are influenced by environmental factors.

## Data Availability

The original contributions presented in the study are included in the article and Supplementary material, further inquiries can be directed to the corresponding author. The sequencing data presented in the study were deposited in the NCBI repository, accession number PRJNA1234064.
