# Supplementary material for: Development of protective immunity against African swine fever depends on host-environment interactions
Source: Front Vet Sci. 2025 Jun 10;12:1553310. doi: 10.3389/fvets.2025.1553310 (PMC12185278; doi:10.3389/fvets.2025.1553310)
Supplement: Supplementary file 1 [file Image_1.pdf]

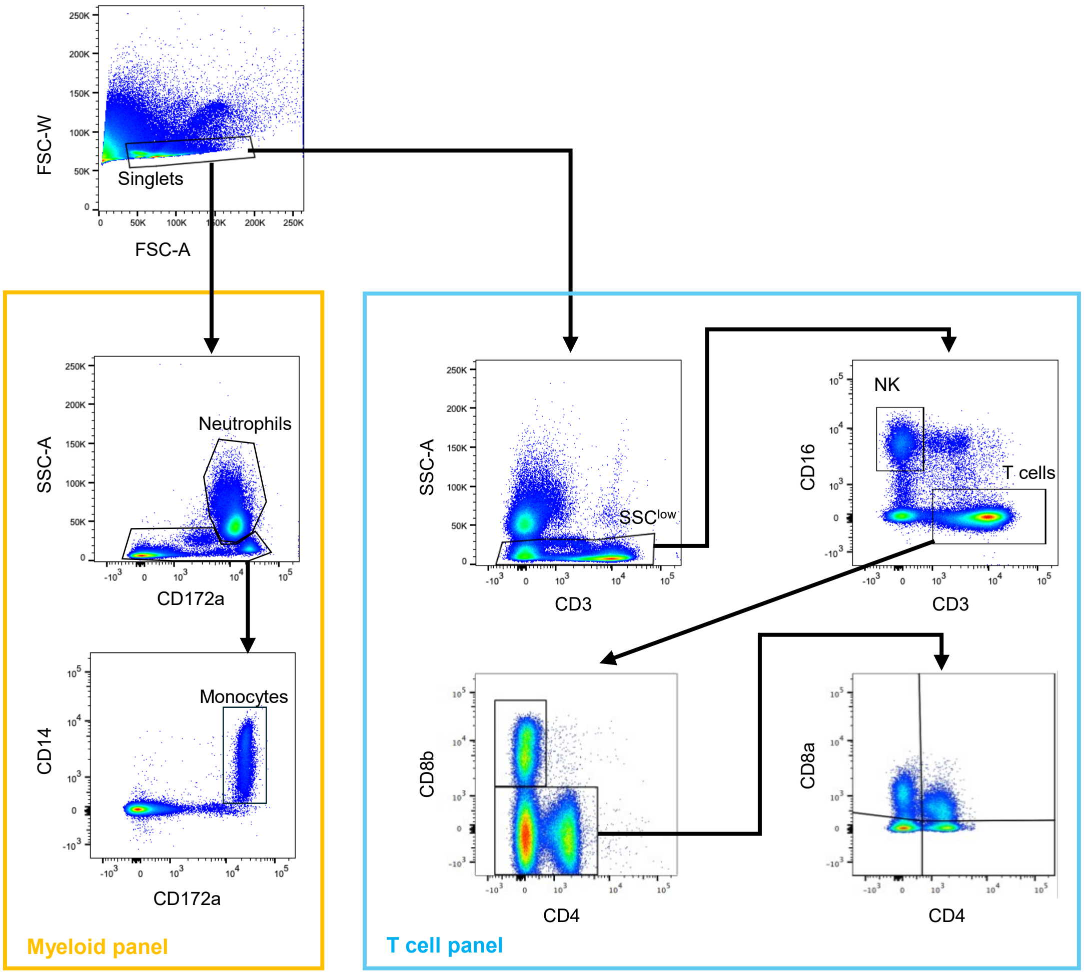

Supplementary Figure 1. Gating strategy. Representative dot plots illustrating the gates used for flow cytometry analysis of leukocyte subsets in whole blood.
