# Supplementary material for: Development of protective immunity against African swine fever depends on host-environment interactions
Source: Front Vet Sci. 2025 Jun 10;12:1553310. doi: 10.3389/fvets.2025.1553310 (PMC12185278; doi:10.3389/fvets.2025.1553310)
Supplement: Supplementary file 2 [file Image_2.pdf]

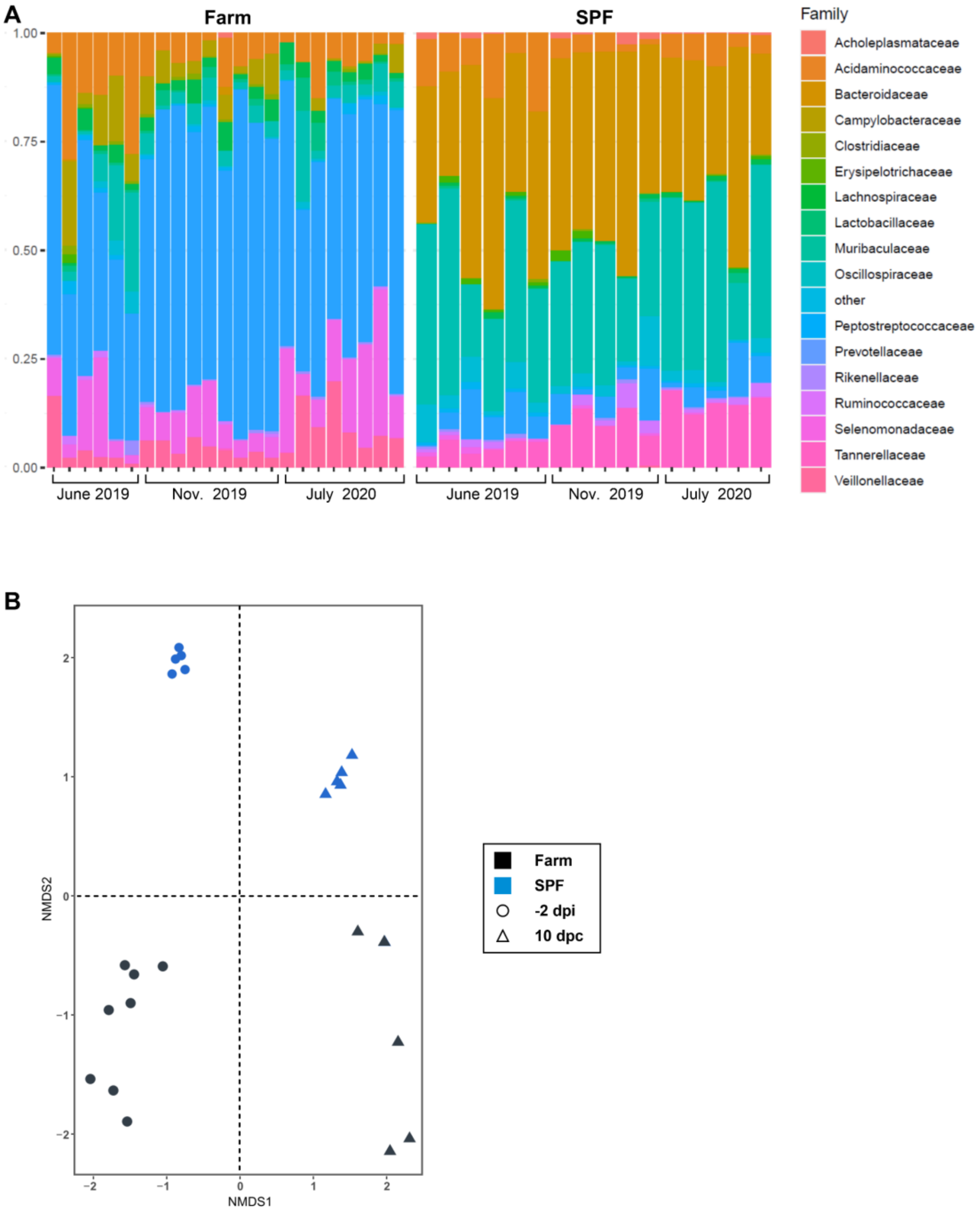

Supplementary Figure 2. Gut microbiota analysis. (**A**) Relative abundance of the most frequent bacterial families at steady state in farm and SPF pigs prior to infection (- 2 dpi) in this study (July 2020) and previous studies (June and November 2019). The SPF pigs were born and raised at the SPF facility of the IVI (n=16) and the farm pigs were from one conventional fattening pig farm in Switzerland (n=23). Each bar represents the data for one animal. (**B**) Non-metric multidimensional scanning (NMDS) ordination of gut microbiome of pigs of this study before infection with the ASFV Estonia 2014 strain (-2 dpi; circles) and 10 dpc with Armenia 2008 (10 dpc; triangles). Each data point represents the value for one animal.
